# Energy Metabolism Dysregulation in Myocardial Infarction: An Integrative Analysis of Ischemic Cardiomyopathy

**DOI:** 10.2174/0115701611289159240724114844

**Published:** 2024-07-26

**Authors:** Zongtao Wang, Zhixin Xie, Tudi Li, Rong Chen, Zhihuan Zeng, Jun Guo

**Affiliations:** 1 Department of Cardiology, The First Affiliated Hospital of Jinan University, Guangzhou, 510630, China;; 2 Department of Cardiology, The First Affiliated Hospital of Guangdong Pharmaceutical University, Guangzhou, 510080, China

**Keywords:** Myocardial infarction, energy metabolism dysregulation, metabolites analysis, functional enrichment analysis, ischemic cardiomyopathy, mitochondria

## Abstract

**Background:**

Myocardial metabolism is closely related to functional changes after myocardial infarction (MI).

**Objective:**

This study aimed to present an integrative examination of human ischemic cardiomyopathy.

**Methods:**

We used both GSE121893 single-cell suspension sequencing and GSE19303 transcription microarray data sets from the GEO database, along with a murine MI model for full-spectrum metabolite detection. Through a systematic investigation that involved differential metabolite identification and functional enrichment analysis, we shed light on the pivotal role of energy metabolism dysregulation in the progression of MI.

**Results:**

Our findings revealed an association between the core regulatory genes CDKN1A, FOS, ITGB4, and MAP2K1 and the underlying pathophysiology of the disease. These genes are identified as critical elements in the complex landscape of myocardial ischemic disorder, highlighting novel insights into therapeutic targets and the intricate biological mechanisms involved.

**Conclusion:**

This analysis provides a framework for future research on the metabolic alterations associated with MI.

## 1 INTRODUCTION

Myocardial Infarction (MI) is a significant global health issue. It occurs when blood flow to the heart is severely reduced or stopped, leading to irreversible damage to the heart muscle [1]. Post-MI, the heart undergoes a series of complex alterations, including metabolic remodeling, a process that has become a focal point of research. The heart primarily relies on fatty acids (60-70%) and glucose (30-40%) for its energy needs. Oxidative phosphorylation in mitochondria is crucial for generating ATP [2, 3]. During MI, ischemia alters substrate availability, enzymatic activities, and mitochondrial function. There is a shift from fatty acid oxidation to glycolysis, leading to inefficient energy production. Mitochondrial dysfunction post-MI contributes to oxidative stress, apoptosis, and further injury. Research on mitochondrial-targeted therapies is gaining momentum [2, 3]. Post-MI, the heart exhibits a shift from fatty acid oxidation to glucose utilization, a phenomenon known as metabolic flexibility. This switch is initially adaptive but becomes maladaptive, leading to heart failure [1]. Key enzymes involved in glycolysis, fatty acid oxidation, and oxidative phosphorylation are altered post-MI. Understanding these changes offers insights into potential therapeutic targets. Here, metabolic remodeling affects contractile function, leading to impaired heart function over time.

Metabolomics is emerging as a powerful tool for identifying metabolic signatures post-MI. It offers the potential for early diagnosis, prognosis, and targeted treatment [4]. Metabolic enzymes, substrates, and intermediates serve as promising biomarkers to assess the risk, progression, and therapeutic response in patients with MI [5, 6]. Pharmacological agents that modulate key metabolic pathways are under investigation, including inhibitors of fatty acid oxidation and stimulators of glucose oxidation [7, 8]. Personalized approaches, considering individual metabolic profiles, are being explored to provide targeted and effective treatment for MI [1]. The field has great potential for exploration, with a growing interest in metabolic pathways, new technologies for analysis, and targeted interventions. Challenges remain in translating laboratory findings into clinical practice [2, 3].

The relationship between MI and metabolic remodeling is complex and multifaceted. Understanding this relationship offers promising avenues for diagnostic and therapeutic innovations.

## 2 MATERIALS AND METHODS

### 2.1 Data Retrieval and Initial Preprocessing

The GSE121893 dataset, a single-cell sequencing study, was downloaded from the National Center for Biotechnology Information (NCBI) Gene Expression Omnibus (GEO; https://www.ncbi.nlm.nih.gov/geo/query/acc.cgi) database [9, 10]. This was carried out using the GEOquery package in R [9]. Upon retrieval, raw count matrices and metadata were extracted and processed. Low-quality cells, defined by a high percentage of mitochondrial gene expression or an extremely low or high number of expressed genes, were filtered out using the scatter package in R [11].

### 2.2 Quality Control and Normalization

Quality control is essential to ensure that the data are reliable and free from artifacts. The raw sequencing data were assessed using FastQC for overall quality, adapter content, and overrepresented sequences [12]. Additionally, the scatter package provided tools for visualizing single-cell RNA-seq data quality metrics. Following the quality assessment, data normalization was carried out using the SCTransform method in the Seurat package [13, 14]. This normalization method regresses unwanted variation and maintains biological heterogeneity.

### 2.3 Dimensionality Reduction

Dimensionality reduction techniques were applied to the normalized data, facilitating the visualization and interpretation of high-dimensional single-cell RNA-seq data [12]. Two methods were applied: t-SNE and UMAP [12]. t-SNE was implemented using the RunTSNE function, while UMAP was applied using the RunUMAP function in the Seurat package [13, 14]. These techniques enabled the identification of meaningful structures within the data, reflecting underlying biological processes.

### 2.4 Clustering and Identification of Marker Genes

The next step was to identify cell clusters and the corresponding marker genes. Graph-based clustering was performed using the FindClusters function in Seurat, relying on a shared nearest neighbor (SNN) modularity optimization-based clustering algorithm. Following clustering, marker genes for each cluster were identified using the FindAllMarkers function in Seurat. The function was applied with default parameters, identifying genes that were differentially expressed in each cluster [13, 14]. The differentially expressed genes (DEGs) were identified using standard methods, such as the DESeq2 package or edgeR package in R, based on criteria with B-H adjusted *p*-value < 0.05 and log2|fold-change| more than 0.25.

### 2.5 Functional Annotation and Pathway Analysis

Functional annotation provides biological context to the identified marker genes. Using the clusterProfiler package in R, Gene Ontology (GO) and Kyoto Encyclopedia of Genes and Genomes (KEGG) pathway enrichment analyses were performed [15]. The key functional roles and biological pathways associated with different cell clusters were identified with the B-H adjusted cut-off *p*-value of 0.05 [11].

### 2.6 Gene Set Enrichment Analysis (GSEA) and Gene Set Variation Analysis (GSVA)

Both GSEA and GSVA are powerful techniques to interpret gene expression data through the lens of previously defined gene sets, revealing underlying biological pathways and processes. The raw expression data underwent normalization, conversion, and proper gene ID mapping to ensure compatibility with gene set databases [16, 17]. Of GSEA, the analysis was performed using the R package clusterProfiler. A pre-ranked gene list was created based on the log2 fold changes, and specific parameters, including the chosen gene sets, number of permutations, and size restrictions, were carefully defined. The Molecular Signatures Database (MSigDB; https://www.gsea-msigdb.org) collections were often utilized in this context [16, 17]. In addition, enriched pathways were graphically represented through enrichment plots, providing a concise visualization of the most significant pathways. A systematic interpretation of the key pathways was then conducted to unravel the underlying biological processes.

For the GSVA analysis, similar to GSEA, expression data were normalized and log-transformed to prepare them for GSVA. Specific collections of gene sets, such as the Hallmark gene set, were selected based on the GSVA method. The parameters of the method, including minimum and maximum gene set sizes, were customized to suit the analysis requirements [18, 19]. Subsequently, the enrichment scores obtained from GSVA were rigorously analyzed for statistical significance, employing suitable statistical methods tailored to the data's characteristics.

### 2.7 Metabolic Analysis

Male C57BL/6 mice aged 8-10 weeks and weighing 20-25g were obtained from SPF (Beijing) Biotechnology Co., Ltd. to study left ventricular myocardial metabolites following MI, with a sample size of 6; control (n *=* 3), MI (n *=* 3). The left anterior descending artery (LAD) was ligated under anesthesia with ketamine and xylazine to induce MI. Tissue collection and sample preparation were performed following previously described protocols. The samples were analyzed using Liquid Chromatography-Mass Spectrometry (LC-MS) as per previous studies. Statistical analysis was conducted using conventional methods, and the validation process was aligned with established protocols [20, 21]. All procedures were conducted in accordance with national guidelines.

After the collection of mass spectral data, chromatographic peaks were aligned and normalized to total signal intensity, and appropriate scaling and missing value imputation methods were applied. Orthogonal Projections to Latent Structures Discriminant Analysis (OPLS-DA) was then employed, with X variables representing the metabolite features and Y variables representing the class information. Cross-validation methods, permutation testing, Variable Importance in Projection (VIP) score calculation, S-plot, and loadings and scores plots were utilized in the OPLS-DA analysis to interpret relationships and identify significant metabolites [22-24]. The threshold for VIP scores, adjusted p-values, and database matching were used to select differential metabolites, and enrichment analysis tools were further employed to identify affected metabolic pathways. This comprehensive methodology provides a robust approach for analyzing metabolite differences using OPLS-DA, encompassing the entire workflow from sample preparation to advanced statistical modeling, and can be adapted to other metabolomics studies requiring classification and identification of differential metabolites.

Here, the metabolites were selected based on two critical criteria to identify significant differences between experimental conditions [20]. First, metabolites exhibiting a VIP value of 1 or higher were chosen. The VIP value quantifies the impact of a particular metabolite's inter-group variance in the classification and discrimination of samples within the model, signifying that a metabolite with a VIP of 1 or higher is considered statistically significant. Second, metabolites were identified with a Fold Change (FC) that is either 2 or more, or 0.5 or less, between the control and experimental groups. This fold change represents a discernible alteration in the metabolite level, reflecting a marked difference when exceeding 2-fold or falling below half. Together, these two parameters provide a stringent and comprehensive approach for pinpointing metabolites that exhibit substantial variations, thereby contributing to the nuanced understanding of underlying biochemical mechanisms.

## 3 RESULTS

### 3.1 scRNA Analysis of Left Ventricular Tissue in Human Ischemic Cardiomyopathy

**Fig. (1):**
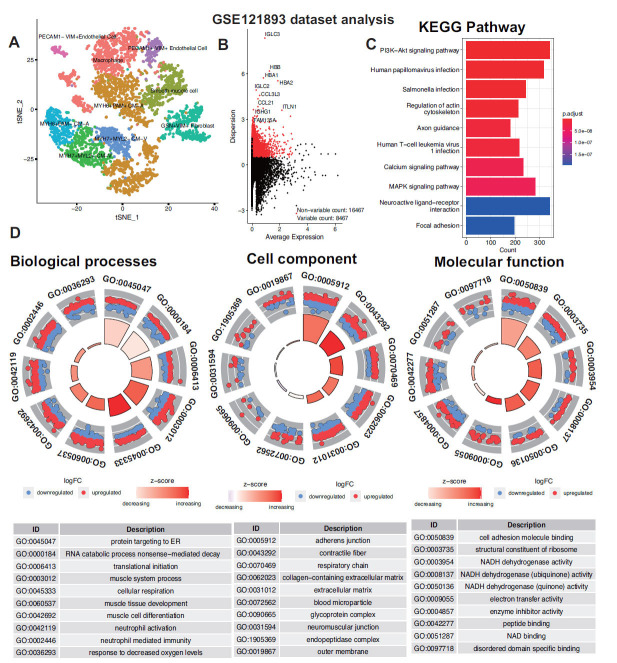
scRNA analysis of highly variable genes: Cell-subgroup classification, expression distribution, and functional enrichment in the context of cell biology. (**A**) Illustration of tSNE (t-Distributed Stochastic Neighbor Embedding) analysis, categorizing the single-cell suspension sequencing data into 9 distinct cell subgroups. (**B**) Representation of the expression distribution of highly variable genes as determined through single-cell sequencing analysis. (**C**) Visualization of the KEGG (Kyoto Encyclopedia of Genes and Genomes) functional enrichment results for the highly variable genes. (**D**) Depiction of the GO (Gene Ontology) functional enrichment analysis results for the genes showing high variability in expression.

In our preliminary analysis, we leveraged the R programming language to mine the single-cell sequencing data of the left ventricular tissue from human ischemic cardiomyopathy transplant hearts available in the GEO database under accession number GSE121893. A total of 4362 cells were retrieved, culminating in the identification of 9 distinct cell subclusters (Fig. **1A**). Further examination led to the discovery of 8467 highly variable genes, including IGLC3, IGLC2, and CCL3L3, among others (Fig. **1B**). Utilizing KEGG functional enrichment analysis on these highly variable genes, we found significant enrichment in pathways, such as the PI3K-Akt signaling pathway, human papillomavirus infection, and the MAPK signaling pathway (Fig. **1C**). Concurrently, GO analysis provided insights into the underlying biological mechanisms, with biological processes, such as protein targeting ER, RNA catabolic process nonsense-mediated decay, and translational initiation, standing out as the most remarkably enriched. Notably, cell component categories, such as adherens junction, contractile fiber, and respiratory chain, exhibited significant differences. Additionally, molecular functions, including cell adhesion molecule binding, structural constituent of ribosome, and NADH dehydrogenase activity, were identified as the primary enriched pathway clusters (Fig. **1D**). These findings present a multifaceted view of the transcriptional landscape of human ischemic cardiomyopathy transplant hearts. By identifying key pathways and biological functions, the study uncovers potential mechanisms of energy metabolism and immune inflammation disorders underlying the pathology, offering valuable insights that may guide future research and therapeutic interventions.

### 3.2 Metabolic Alterations in the MI Development

**Fig. (2):**
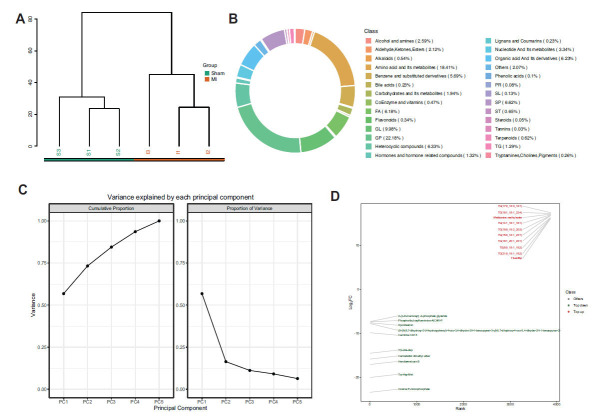
Unveiling the metabolic landscape in mouse MI: Analysis of unsupervised clustering, metabolite identification, and principal component exploration. (**A**) Illustrates the unsupervised clustering of metabolic detection in the construction of a mouse MI model, highlighting the differences between the MI model group and the sham surgery group. (**B**) Depicts the identification analysis of different metabolite components, distinguishing a total of 28 types of metabolite components. (**C**) Represents the principal component analysis of widely targeted metabolic products. (**D**) Showcases the main differential metabolic products that are upregulated and downregulated.

In our initial study constructing a mouse MI model, we discovered significant differences between the MI model group and the sham surgery group through unsupervised clustering (Fig. **2A**). A detailed analysis of different metabolic components led to the identification of 28 distinct metabolite classes. These classes were predominantly composed of amino acids and their metabolites, Fatty Acids (FA), Glycerophospholipids (GP), Sphingolipids (SP), and Glycolipids (GL) (Fig. **2B**). Principal Component Analysis (PCA) revealed that the metabolites distinguishing the myocardial infarction tissues were primarily concentrated in the first and second principal components (Fig. **2C**). This clustering pattern provides evidence for the distinct metabolic alterations associated with MI.

Differential analysis further highlighted a total of 1630 distinct metabolic products that varied between the control and myocardial infarction groups. Among these, 779 were downregulated metabolites, and 851 were upregulated. The upregulated metabolic products primarily included compounds, such as TG (17:0_18:0_18:1), TG (18:1_18:1_22:4), and Methionine methyl ester. Conversely, downregulated metabolic products were chiefly characterized by Hemibrevetoxin B, Trp-Arg-Met, and Inosine 5'-monophosphate (Fig. **2D**). Here, these results provide a comprehensive understanding of the metabolic alterations in the mouse MI model. The specific changes in metabolite levels highlight potential metabolic pathways and biomolecules that may be implicated in the pathology of MI.

### 3.3 Differential Metabolite Analysis in MI Development

**Fig. (3):**
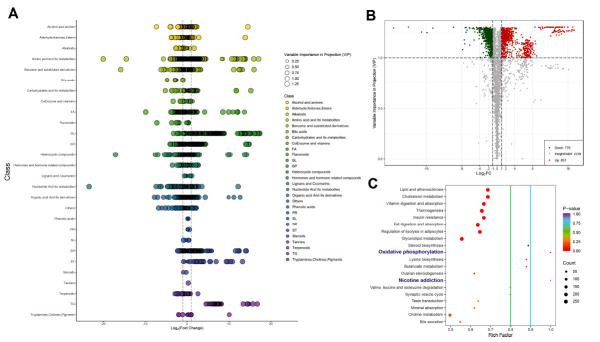
Comprehensive analysis of differential metabolites in mouse myocardial tissue post-infarction: VIP, expression changes, and functional enrichment. (**A**) Illustrates the Variable Importance in Projection (VIP) and differential Fold Change expression of metabolites; (**B**) A volcano plot highlighting the predominantly upregulated changes in metabolites in the myocardial tissue of mice post-myocardial infarction; (**C**) Presents the results of the functional enrichment analysis of differential metabolites.

Through the visualization of VIP and FC in differential metabolite analysis, several patterns emerged. Metabolites categorized under Triglycerides (TG), organic acid and its derivatives, and Glycolipids (GL) were mainly observed to be upregulated. In contrast, metabolites classified as nucleotides and their metabolites, bile acids and flavonoids were predominantly downregulated (Fig. **3A**). A volcano plot further reinforced these findings, illustrating that the metabolic alterations in the myocardial tissues of mice post-MI were characterized mainly by upregulation (Fig. **3B**). This shift in the metabolite profile provides insights into the potential regulatory mechanisms underpinning MI. Differential metabolite functional enrichment analysis sheds light on the significant pathways in which these differentially regulated metabolites participate. Remarkably, the metabolites were found to be primarily involved in the processes of oxidative phosphorylation, nicotine addiction, and valine, leucine, and isoleucine degradation (Fig. **3C**).

### 3.4 Detection and Functional Enrichment of Hub Regulators

**Fig. (4):**
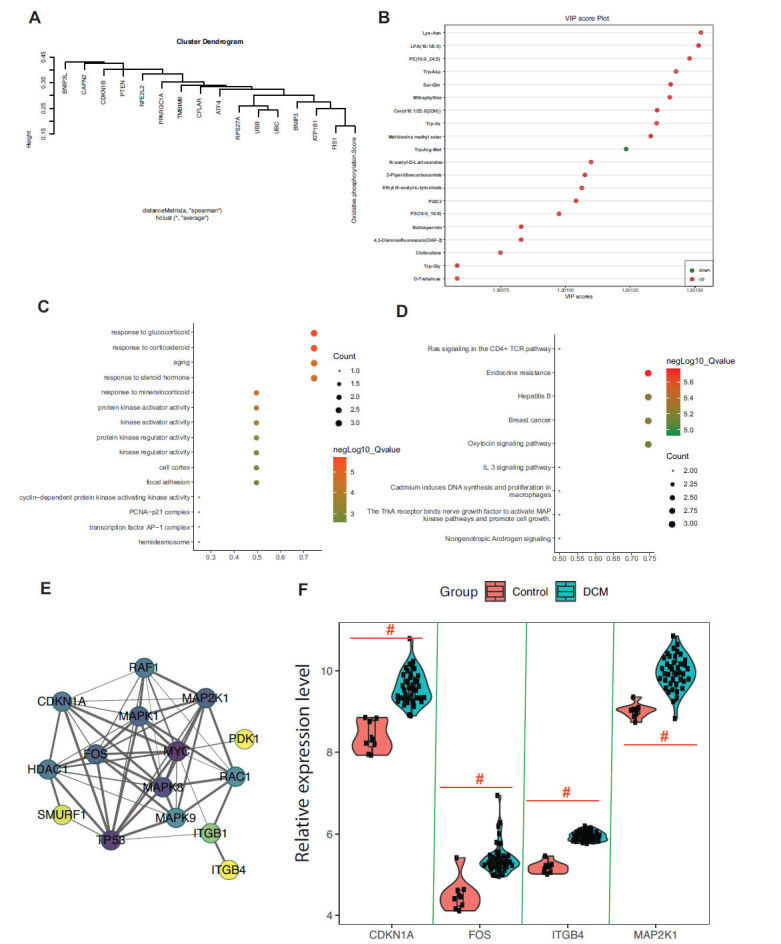
Integration of transcriptomic and metabolomic insights into energy metabolism; Analysis of key genes and pathways in the GSE19303 dataset. (**A**) Shows the association analysis between the expression levels of 15 significantly differentially expressed energy metabolism-related genes and the GSVA scores of the oxidative phosphorylation pathway in the GSE19303 data. (**B**) Illustrates the main energy metabolites identified in metabolomics. (**C** and **D**) Represent the pathway enrichment analysis results for the 15 core energy metabolism-related genes. (**E**) Depicts the Protein-Protein Interaction (PPI) analysis of the core genes. (**F**) Displays the expression of the core regulatory genes CDKN1A, FOS, ITGB4, and MAP2K1.

**Fig. (5):**
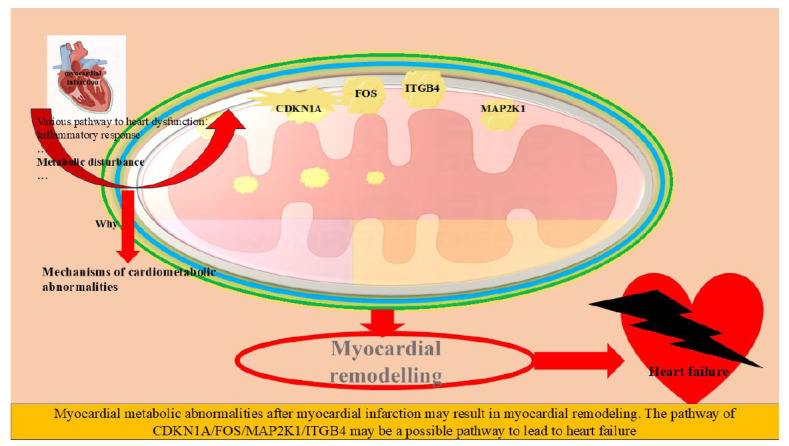
The new pathway of metabolism-related core regulatory genes CDKN1A, FOS, ITGB4, and MAP2K1 is a promising foundation for gaining a deeper understanding of the complex interplay between energy metabolism and MI.

The analysis of data from the GEO database, specifically GSE19303, which consists of 8 normal myocardial tissues and 49 ischemic heart disease tissues, reveals intricate insights into the pathophysiology of MI. There were 15 energy metabolism-related genes that exhibited significant expression differences between normal and ischemic myocardial tissues. The expression levels of these genes were significantly correlated with the GSVA scores of the oxidative phosphorylation pathway, possibly elucidating alterations in energy metabolism in ischemic heart disease tissues (Fig. **4A**). Metabolically, Lys-Asn, LPA (18:1/0:0), and PE (18:0_24:5) were identified as the major upregulated energy metabolites. These changes in compounds might reflect critical shifts in metabolic processes within myocardial ischemic disease (Fig. **4B**). Fifteen differentially expressed energy metabolism-related genes were primarily enriched in steroid-responsive pathways, such as response to glucocorticoids, corticosteroids, and mineralocorticoids (Figs. **4C** and **D**). These signaling pathways might play vital roles in the underlying biology of ischemic heart disease. In addition, PPI interaction and differential expression analysis revealed CDKN1A (Cyclin-Dependent Kinase Inhibitor 1A), ITGB4 (Integrin Subunit Beta 4), MAP2K1 (Mitogen-Activated Protein Kinase Kinase 1), and FOS (Fos Proto-Oncogene, AP-1 Transcription Factor Subunit) as the primary regulatory genes (Figs. **4E** and **F**). These findings suggest that these genes may serve as crucial regulatory factors in energy metabolism during the progression of MI (Fig. **5**).

## 4 DISCUSSION

MI is a world health problem that leads to myocardial dysfunction and heart failure [25]. Among the various mechanisms, metabolic disorders in myocardial cells are one of the important mechanisms. Mitochondria, the energy production center within cardiac cells, play a crucial role in cardiac function. Post-MI, cardiac mitochondrial function is impaired, resulting in metabolic abnormalities that impact the normal function of myocardial cells [26].

Metabolomics is the systematic study of small molecule metabolites within a biological system [27]. Analytic techniques like mass spectrometry (MS) and nuclear magnetic resonance (NMR) spectroscopy enable the simultaneous measurement of thousands of metabolites, providing a snapshot of metabolic status [28]. In applications of cardiovascular research, metabolomics has uncovered biomarkers that offer superior sensitivity and specificity for early diagnosis and risk stratification in diseases, such as heart failure, MI, and hypertension. Metabolic profiling unravels the intricate metabolic pathways involved in CVDs, such as lipid metabolism, glucose utilization, and mitochondrial dysfunction [27]. This understanding paves the way for targeted therapeutic interventions. Nutritional interventions and their impact on cardiovascular health can be precisely assessed using metabolomics. In personalized therapy, pharmacometabolomics analyzes metabolic responses to therapeutic interventions, offering opportunities for personalized medicine in CVDs [6, 29-31]. This helps optimize drug efficacy and minimize adverse reactions. In addition, combining metabolomics with genomics, proteomics, and transcriptomics (multi-omics approach) enables a comprehensive understanding of CVDs at various biological levels. Such integration offers an unparalleled view of disease mechanisms.

In the present study, we have found a novel association between energy metabolism-related core regulatory genes (Fig. **5**), CDKN1A, FOS, ITGB4, and MAP2K1, and the progression of MI, one of the most critical forms of CVD. The following discussion explores the relationship between these genes and cardiovascular ailments, with a particular focus on MI. CDKN1A, a crucial cell cycle regulator, plays an essential role in cell growth, DNA repair, and apoptosis. Recent studies have linked alterations in CDKN1A expression to cardiac hypertrophy and heart failure [32, 33]. Its role in energy metabolism and oxidative stress response has begun to elucidate potential pathways through which CDKN1A may contribute to MI [32, 33]. Further research on CDKN1A's control over cellular energy homeostasis may provide insights into potential therapeutic targets for preventing or alleviating MI.

FOS is a component of the activator protein-1 (AP-1) complex, a crucial player in various cellular processes, including differentiation, proliferation, and apoptosis. FOS has been shown to be activated in response to cardiovascular stress, such as hypertension [34-36]. Its dysregulation in MI suggests a potentially critical role in the pathological remodeling of cardiac tissue following ischemic injury. Understanding the specific mechanisms by which FOS regulates cardiac response to stress can guide targeted therapies for ischemic heart diseases, including MI [34-36].

ITGB4 is a part of the integrin family involved in cell adhesion, migration, and signaling. Previous research has emphasized its role in vascular biology, particularly in angiogenesis. The discovery of its association with MI progression signifies its potential importance in vascular remodeling and response to ischemic injury. Targeting ITGB4-mediated pathways might offer new avenues for enhancing revascularization and healing following MI [37, 38].

MAP2K1 is involved in the MAPK signaling pathway, a key regulator of cellular growth, differentiation, and stress response. Dysregulation of MAP2K1 has been associated with various CVDs, including heart failure and arrhythmias. Its relationship with energy metabolism and connection to MI adds another layer of complexity to its role in cardiovascular health [39, 40]. Further understanding of how MAP2K1 modulates energy metabolic pathways in the heart may offer innovative therapeutic strategies for MI patients.

Strong correlations between metabolites indicate that these metabolites lie along the same or related metabolic pathways and are, therefore, co-regulated. A series of achievements have been made in understanding the myocardial metabolism following MI, with regard to mechanisms, such as oxidative stress [26, 41], histidine triad nucleotide-binding protein 2 (HINT2) [42], and reactive oxygen species (ROS) [43]. They have provided insights into the post-MI metabolic changes. Although we can understand the mechanisms of metabolic abnormalities through laboratory studies, translating these findings into effective treatment strategies remains a challenge in clinical practice.

## CONCLUSION

The identification of CDKN1A, FOS, ITGB4, and MAP2K1 as core regulatory genes in energy metabolism associated with MI opens exciting new paths in cardiovascular research. This study provides a promising foundation for gaining a deeper understanding of the complex interplay between energy metabolism and MI. Investigating the specific roles, interactions, and pathways of these genes in the heart will be vital for developing targeted interventions to improve outcomes for patients suffering from MI and other related cardiovascular conditions. Continued research in this direction may hold the key to unlocking novel, effective therapeutic strategies that leverage the intricate connections between cellular energy regulation and cardiovascular health.

## LIMITATIONS

There are still some interesting questions that need to be addressed in the future. First, what is the relationship among CDKN1A, FOS, ITGB4, and MAP2K1 in metabolic disorders after MI. Second, how do changes in these genes cause changes in metabolites after MI, and what is the relationship among metabolites, such as Lys-Asn, LPA (18:1/0:0), and PE, after MI?

## Data Availability

All the data and supporting information are provided within the article.

## LIST OF ABBREVIATIONS

ATP: Adenosine Triphosphate
CDKN1A: Cyclin-Dependent Kinase Inhibitor 1A
DEGs: Differentially Expressed Genes
GEO: Gene Expression Omnibus
HINT2: Histidine Triad Nucleotide-binding Protein 2
ITGB4: Integrin Subunit Beta 4
MAP2K1: Mitogen-Activated Protein Kinase Kinase 1
MI: Myocardial Infarction
NCBI: National Center for Biotechnology Information
QC: Quality Control
ROS: Reactive Oxygen Species
t-SNE: t-Distributed Stochastic Neighbor Embedding
UMAP: Uniform Manifold Approximation and Projection
